# Association between *SRD5A2* rs523349 and rs9282858 Polymorphisms and Risk of Benign Prostatic Hyperplasia: A Meta-Analysis

**DOI:** 10.3389/fphys.2017.00688

**Published:** 2017-09-12

**Authors:** Xian-Tao Zeng, Xin-Jun Su, Sheng Li, Hong Weng, Tong-Zu Liu, Xing-Huan Wang

**Affiliations:** ^1^Department of Urology, Zhongnan Hospital of Wuhan University Wuhan, China; ^2^Center for Evidence-Based and Translational Medicine, Zhongnan Hospital of Wuhan University Wuhan, China

**Keywords:** *SRD5A2*, BPH, susceptibility, polymorphism, risk

## Abstract

**Objective:** Previous studies have reported that rs523349 (V89L) and rs9282858 (A49T) polymorphisms in the gene 5α-reductase II (*SRD5A2*) are associated with the risk of benign prostatic hyperplasia (BPH), but different opinions have emerged. In view of distinct discrepancies among those findings, we performed this meta-analysis to ascertain a more accurate association between *SRD5A2* rs523349 and rs9282858 polymorphisms and the risk of BPH.

**Methods:** Studies investigating the association between *SRD5A2* rs523349 and rs9282858 polymorphisms and susceptibility to BPH were searched from the databases of PubMed, Embase, Wanfang, and Chinese National Knowledge Infrastructure (CNKI).The strength of correlation was assessed by crude odds ratios (ORs) with their corresponding 95% confidence intervals (95% CIs). Moreover, subgroup analysis was conducted to further ascertain such relationship and investigate sources of heterogeneity.

**Results:** *SRD5A2* rs9282858 (A49T) polymorphism showed a significant correlation with increased BPH susceptibility under allele T vs.allele A genetic model (OR = 2.51, 95% CI = 1.29–4.88) in total analysis, and stratification analysis by ethnicity also revealed a similar association in Caucasian group under the same contrast. *SRD5A2* rs523349 (V89L) polymorphism showed no significant role in BPH occurrence in total analysis, but its reducing and increasing effects on the disease risk were reflected in Caucasian and other-ethnicity subgroups, respectively, after stratification analysis by ethnicity.

**Conclusion:** In conclusion, *SRD5A2* rs9282858 polymorphism may elevate the susceptibility to BPH, while the polymorphism rs523349 may exert different influences on the disease in people of different ethnic lines.

## Introduction

Benign prostatic hyperplasia (BPH) is one of the most common diseases in men over 60 years old in western world (Ziada et al., 1999), and its incidence has been reported to increase with age, thus dramatically contributing to the burden of aging society (van Rij and Gilling, 2015). BPH is characterized clinically by prostatic enlargement and lower urinary tract symptoms (LUTS) and histologically by hypertrophy and hyperplasia of the prostatic cells, and may lead to dysuria, frequency of urination, urinary urgency, nocturia, and various complications such as bladder stones, urinary tract infection, and hydronephrosis, which can bring great agony to the patients (Choubey et al., 2015; Wu et al., 2016). Statistics show that 6% of the global population suffer from this disease as of 2010 (Vos et al., 2012). Although this disorder has not been confirmed to have significant correlation with the cancer risk, it does adversely affect the life quality of patients (Chang et al., 2012). The exact pathogenesis of BPH remains elusive, but this multifactorial disease may involve environmental, hormonal, and genetic factors in its etiology (Izmirli et al., 2011).

Androgens, mainly including testosterone and dihydrotestosterone (DHT), have been suggested to be involved in the occurrence of BPH (Marcelli and Cunningham, 1999). DHT has apparently higher binding affinity with androgen receptor compared with testosterone (Wilbert et al., 1983), and wields significant impact on prostatic growth and development of male external genitalia (Choubey et al., 2015). The development of BPH in middle-aged men is associated with high levels of DHT, and the effect of DHT on the prostate is hyperplasia followed by the urinary symptoms (Eaton, 2003; Agamia et al., 2016). In addition, clinical response of prostate cancer to anti-androgen therapy can be predicted by DHT levels (Geller et al., 1984). Because DHT, in the prostate, can be converted from testosterone by the enzyme 5α-reductase type 2 encoded by the 5α-reductase II (*SRD5A2)* gene (Wilson et al., 1993), its levels and resulting androgen action in different individuals can be affected by the activity of this enzyme (Rajender et al., 2009). The gene *SRD5A2* is mapped to chromosome 2p23, and has 4 introns and 5 exons (Thigpen et al., 1992). The activity of SRD5A2 varies among people of different ethnic lines, and may be altered by polymorphisms in the gene *SRD5A2* (Ross et al., 1992; Reichardt et al., 1995; Makridakis et al., 2000). The valine to leucine substitution at codon 89 (rs523349) and the alanine to threonine substitution at codon 49 (rs9282858) are reported to have functional consequences (Salam et al., 2005). *In vitro* studies have shown that the rs9282858 (A49T) polymorphism increased the SRD5A2 enzyme activity by about 5-fold, whereas the rs523349 (V89L) polymorphism was associated with approximately 40% lower SRD5A2 activity (Makridakis et al., 1999; Salam et al., 2005). Many researchers have concentrated their attention on the impact of these two polymorphisms on risk of BPH, but controversies still exist.

To better understand the role of these two single nucleotide polymorphisms (SNPs) in BPH susceptibility, this meta-analysis incorporating a total of 1,865 cases and 1,424 controls was carried out.

## Materials and methods

### Search strategy

An extensive literature search was performed in PubMed, Embase, Wanfang, and Chinese National Knowledge Infrastructure (CNKI) databases to identify studies examining the impact of *SRD5A2* rs523349 and/or rs9282858 polymorphisms on risk of BPH using search terms as follows: “*SRD5A2*” or “5α-reductase type 2,” “BPH” or “benign prostatic hyperplasia,” and “polymorphism” or “variant” or “variation” or “SNP.” Bibliographies of all pertinent articles were also manually checked for other relevant articles.

### Eligibility criteria

Eligible studies should accord with the following criteria: (1) containing original data; (2) published in English or Chinese language; (3) including sufficient data on genotype and/or allele distribution in cases and controls; and (4) with a case-control design. Studies were excluded for the following reasons: (1) with no control subjects; (2) not about *SRD5A2* polymorphisms or BPH; and (3) abstract, reviews, case reports, and comments. When overlapping data were contained in more than one publication, the one with the largest number of objects was selected.

### Data extraction

Two authors independently reviewed the included studies and extracted essential data from them. The collected information included: first author's name, publication year, country, ethnicity, source of control, studied polymorphism(s), genotyping method, sample size, genotype and/or allele distribution in both case and control groups, and *P*-value for Hardy-Weinberg Equilibrium (HWE) in controls. Any discrepancies over recorded information were resolved through discussion between the two authors until a final consensus was reached.

### Statistical analysis

STATA software version 12.0 was used to conduct all data syntheses in this meta-analysis. Crude odds ratios (ORs) with their corresponding 95% confidence intervals (95% CIs) were calculated to evaluate the effects of *SRD5A2* rs523349 and rs9282858 polymorphisms on BPH risk. Whether genotype distribution in control group was in accordance with HWE expectation was determined by Chi-square test. Significance of summary ORs was examined using Z test. Heterogeneity between studies was assessed with Chi-Square based Q-statistical test. *P*-value smaller than 0.05 in Q test indicated significant heterogeneity, and random-effects model was applied for OR estimation in this case; otherwise, fixed-effects model was utilized. For *SRD5A2* rs523349 (V89L) polymorphism, this meta-analysis examined the association between allele L and BPH risk compared with that for allele V (L vs. V), homozygote LL was contrasted with VV (LL vs. VV), heterozygote VL was contrasted with VV (VL vs. VV); meanwhile, dominant (LL + VL vs. VV) and recessive (LL vs. VL+VV) models were also used, so was rs9282858 (A49T). Moreover, subgroup analysis was conducted to further ascertain such relationship and investigate sources of heterogeneity. The effect of single studies on overall estimates was evaluated using one-way sensitivity analysis for studied polymorphism(s) on which more than 5 studies concerned. In addition, Begg's funnel plot and Egger's linear regression test were performed for inspecting potential publication bias among included studies, the number of which was more than 5. All *P*-values were two-sided, with *P* < 0.05 suggesting statistical significance.

## Results

### Study characteristics

**Figure 1:**
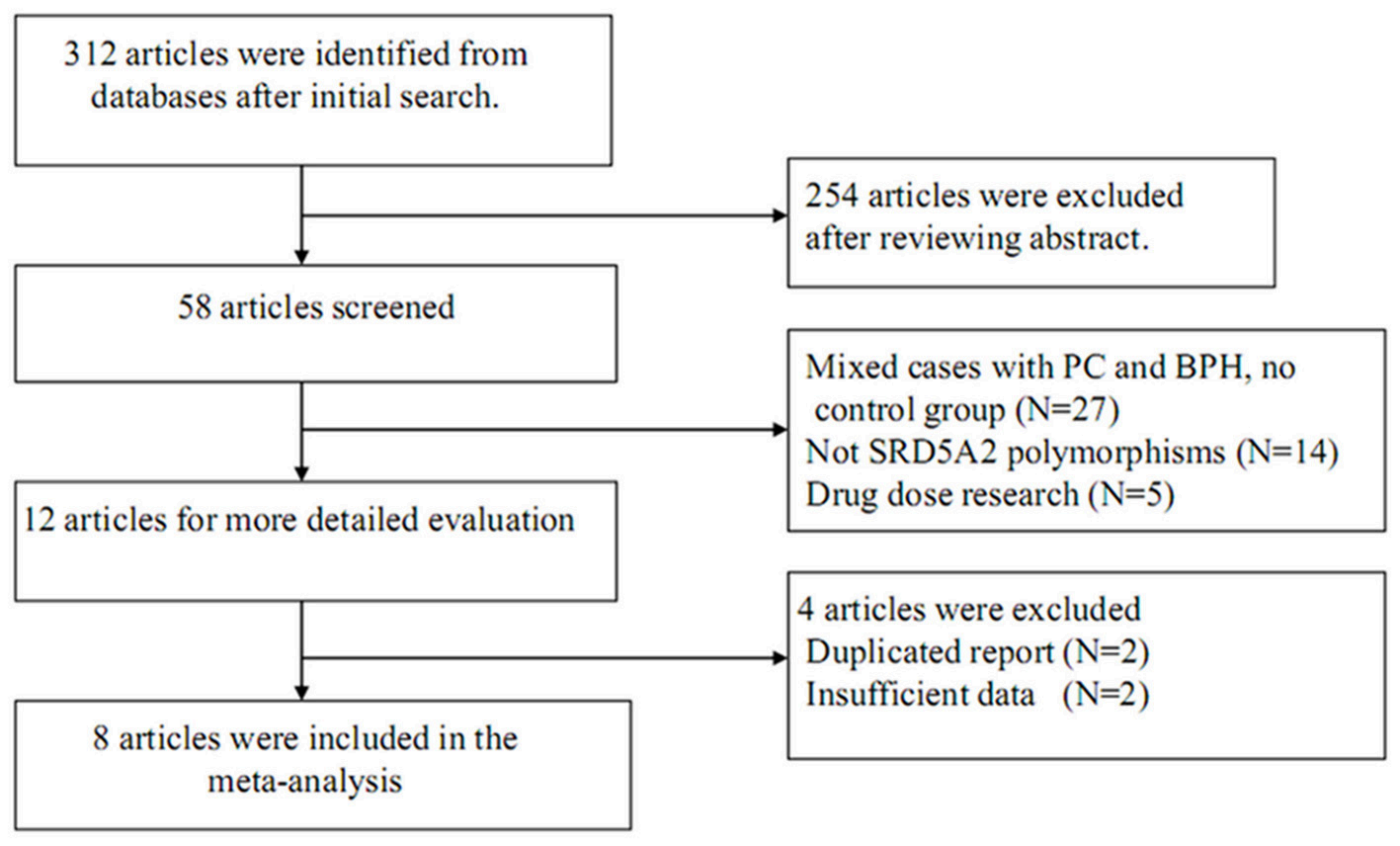
Flow chart of study selection.

The process for study retrieval and selection is described in Figure 1. Initially, a total of 312 articles were identified from the databases. Then, 254 articles were firstly excluded after abstract review. Among the remaining 58 articles, 27 had no control group, 14 were not associated with *SRD5A2* polymorphisms, 5 were drug dose researches, 2 were duplicate reports, and another 2 contained insufficient data. As a result, 1,865 cases and 1,424 controls were included in the present meta-analysis (Li et al., 2003; Giwercman et al., 2005; Salam et al., 2005; Das et al., 2008; Rajender et al., 2009; Izmirli et al., 2011; Choubey et al., 2015; Ersekerci et al., 2015). Characteristics of the included studies are provided in Table 1.

**Table 1:** Major characteristics of studies included in the present meta-analysis.

**First author, year**	**Country**	**Ethnicity**	**No. of cases/controls**	**Case**					**Control**					**HWE**	**Genotyping method**
**rs523349**				**VV**	**VL**	**LL**	**V**	**L**	**VV**	**VL**	**LL**	**V**	**L**		
Ersekerci et al., 2015	Turkey	Caucasian	28/30	16	11	1	43	13	13	15	2	41	19	0.395	PCR-RFLP
Li et al., 2003	Japan	Asian	228/243	64	127	37	255	201	75	117	51	267	219	0.668	PCR-RFLP
Rajender et al., 2009	India	Asian	40/96	12	16	12	40	40	29	42	25	100	92	0.226	PCR-RFLP
Salam et al., 2005	USA	Hispanic	264/44	111	118	35	340	188	25	17	2	67	21	0.675	PCR-SCP
Salam et al., 2005	USA	Caucasian	62/28	36	14	12	86	38	10	17	1	37	19	0.061	PCR-SCP
Salam et al., 2005	USA	mixed	377/109	175	153	49	503	251	50	53	6	153	65	0.091	PCR-SCP
Choubey et al., 2015	India	Asian	188/154	145	43		/	/	104	50		/	/	/	PCR
Das et al., 2008	Singapore	Asian	96/28	21	75		/	/	8	20		/	/	/	PCR
Giwercman et al., 2005	Sweden	Caucasian	45/217	41		4	/	/	184		33	/	/	/	Allele-specific PCR
Izmirli et al., 2011	Turkey	Caucasian	35/29	/	/	/	19	51	/	/	/	22	36	/	PCR-RFLP
**rs9282858**				**AA**	**AT**	**TT**	**A**	**T**	**AA**	**AT**	**TT**	**A**	**T**		
Giwercman et al., 2005	Sweden	Caucasian	45/213	40	5	0	85	5	200	13	0	413	13	0.646	Allele-specific PCR
Rajender et al., 2009	India	Asian	40/96	40	0	0	80	0	96	0	0	192	0	/	PCR-RFLP
Salam et al., 2005	USA	Mixed^*^	382/108	370	11	1	751	13	107	0	1	214	2	/	PCR-SSCP
Izmirli et al., 2011	Turkey	Caucasian	35/29	/	/	/	52	18	/	/	/	53	5	/	PCR-RFLP

HWE, Hardy-Weinberg equilibrium; PCR-RFLP, polymerase chain reaction-restriction fragment length polymorphism; PCR-SCP, PCR-strand conformation polymorphism; PCR-SSCP, PCR-single strand conformation polymorphism;

* *Multiethnic population (including Caucasians, Hispanics, African-Americans, and Asians)*.

### Meta-analysis results

**Figure 2:**
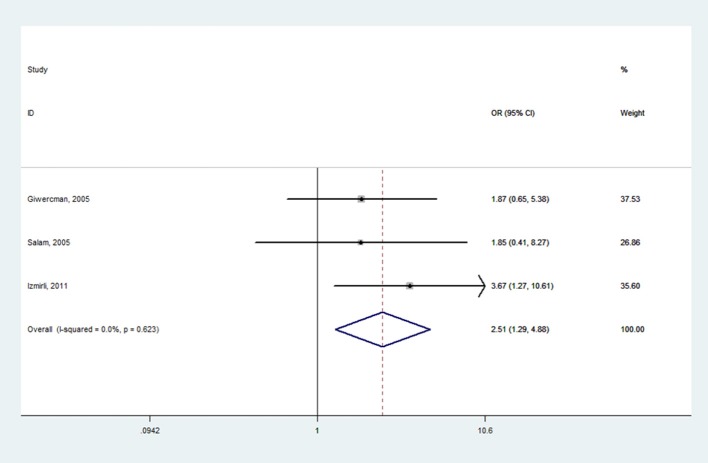
Forest plot for the correlation between *SRD5A2* rs9282858 polymorphism and BPH risk under allele T vs. allele A genetic model.

Table 2 shows the meta-analysis results for the effects of *SRD5A2* rs9282858 and rs523349 polymorphisms on BPH susceptibility. For *SRD5A2* rs9282858 (A49T) polymorphism, an increased BPH risk was observed under allele T vs. allele A genetic contrast in total analysis (OR = 2.51, 95% CI = 1.29–4.88; Figure 2). Since no polymorphism was observed at the A49T polymorphic site in Rajender's study, it was automatically excluded by the analysis software. Additionally, two of the analyzed studies (Giwercman et al., 2005; Salam et al., 2005) have CI that include 1, which indicates that the results from these studies are statistically insignificant. To further ascertain such relationship between *SRD5A2* rs9282858 (A49T) polymorphism and BPH observed in the total analysis (Figure 2), subgroup analysis by ethnicity was conducted and a similar association was observed in Caucasian group under the same contrast (T vs. A: OR = 2.75, 95% CI = 1.32–5.69) (Table 2).

**Table 2:** Meta-analysis results on the relationship of *SRD5A2* rs523349 and rs9282858 polymorphisms with BPH risk.

**SNP**		**Odds ratio (95% confidence interval)/*****P*****-value of heterogeneity test**				
**rs523349**		**LL vs. VV**	**LL+VL vs. VV**	**LL vs. VL+VV**	**allele L vs. allele V**	**VL vs. VV**
Ethnicity	Caucasian	1.52 (0.36, 6.37)/0.210	0.47 (0.24, 0.93)/0.615	1.11 (0.22, 5.48)/0.101	0.99 (0.65, 1.51)/0.234	0.36 (0.14, 0.92)/0.199
	Asian	0.92 (0.57, 1.46)/0.581	0.94 (0.71, 1.23)/0.197	0.84 (0.54, 1.32)/0.286	0.98 (0.78, 1.24)/0.678	1.20 (0.82, 1.75)/0.517
	Other	2.74 (1.27, 5.92)/0.554	1.18 (0.83, 1.68)/0.118	2.72 (1.28, 5.70)/0.796	1.33 (1.01, 1.75)/0.195	1.08 (0.58, 2.00)/0.118
Total		1.32 (0.91, 1.92)/0.168	0.95 (0.77, 1.17)/0.076	1.29 (0.71, 2.35)/0.035	1.10 (0.93, 1.29)/0.276	0.86 (0.55, 1.34)/0.023
**rs9282858**		**TT vs. AA**	**TT**+**AT vs. AA**	**TT vs. AT**+**AA**	**allele T vs. allele A**	**AT vs. AA**
Ethnicity	Caucasian	−/−	1.92 (0.65, 5.70)/−	−/−	2.75 (1.32, 5.69)/0.373	1.92 (0.65, 5.70)/−
	Asian	−/−	−/−	−/−	−/−	−/−
	Mixed	0.29 (0.02, 4.66)/−	3.47 (0.45, 26.99)/−	0.28 (0.02, 4.53)/−	1.85 (0.41, 8.27)/−	6.67 (0.39, 114.17)/−
Total		0.29 (0.02, 4.66)/−	2.34 (0.91, 6.06)/0.604	0.28 (0.02, 4.53)/−	2.51 (1.29, 4.88)/0.623	2.67 (1.00, 7.12)/0.386

**Figure 3:**
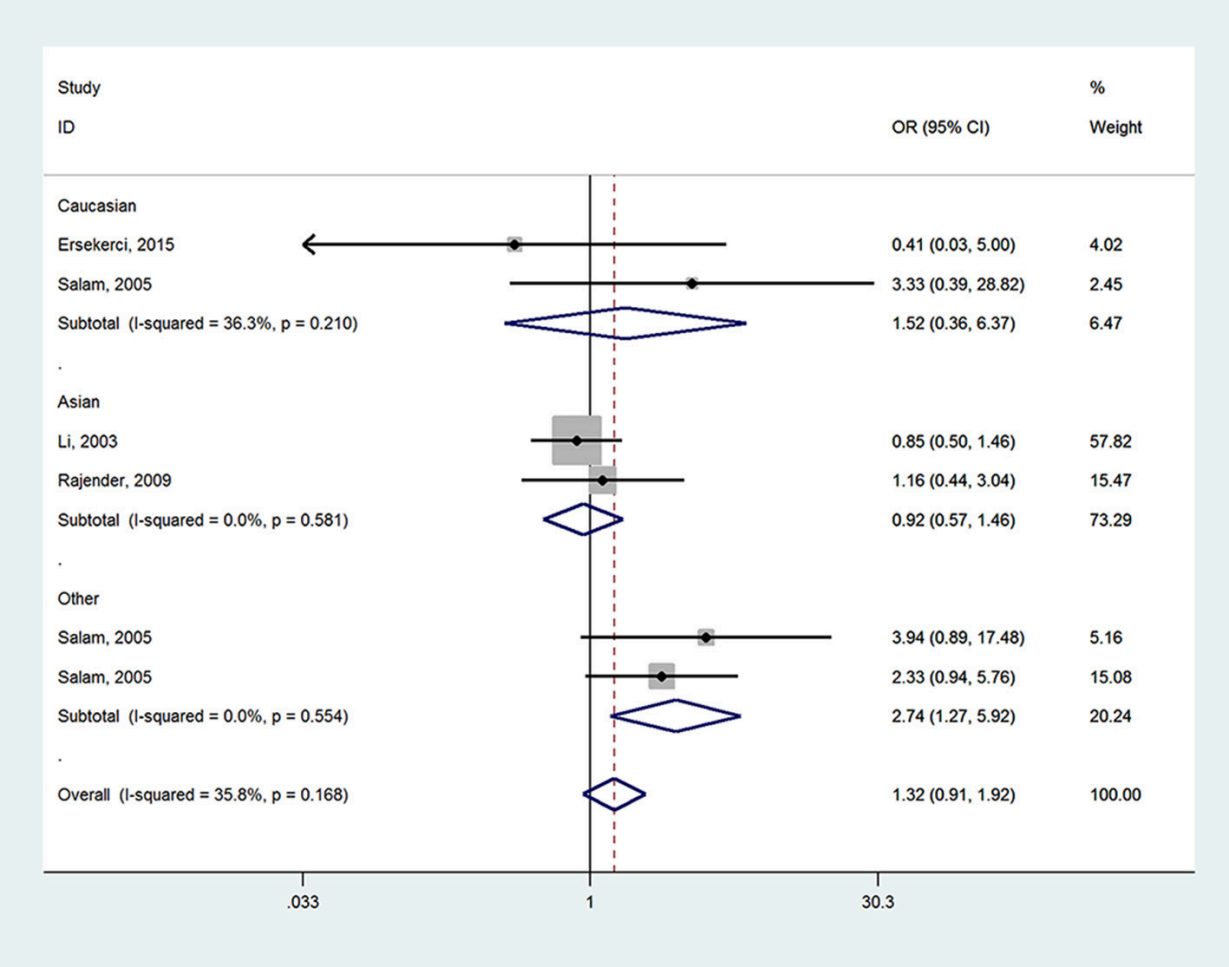
Forest plot for the association between *SRD5A2* rs523349 polymorphism and BPH susceptibility under LL vs. VV model after stratification analysis by ethnicity.

**Figure 4:**
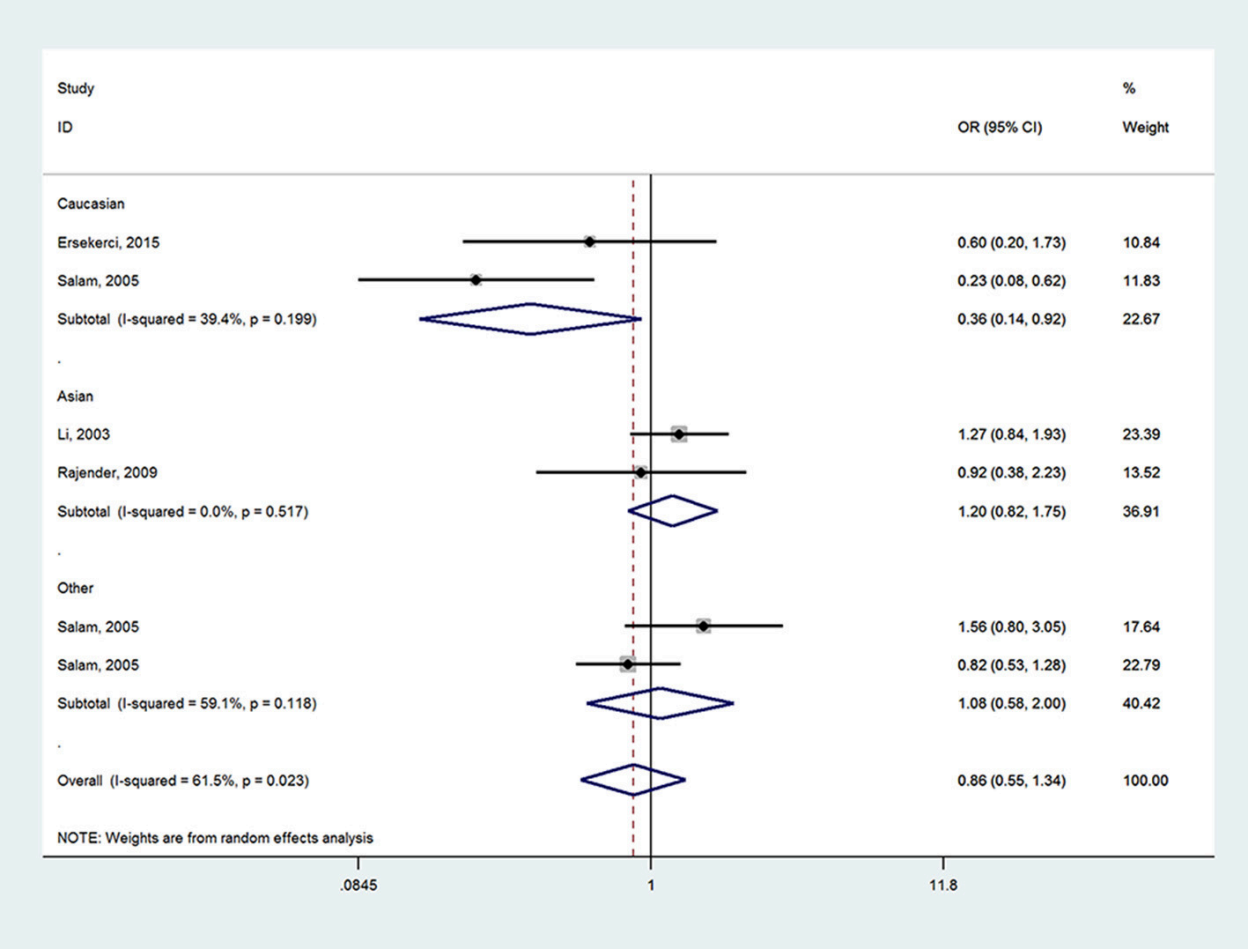
Forest plot for the relationship between *SRD5A2* rs523349 polymorphism and BPH susceptibility under VL vs. VV model after stratified analysis by ethnicity.

When it came to *SRD5A2* rs523349 (V89L) polymorphism, no significant relationship was detected with BPH risk in total analysis. However, after the subgroup analysis by ethnicity, a positive association there between was revealed in other-ethnicity group under LL vs. VV (OR = 2.74, 95% CI = 1.27–5.92) (Figure 3), LL vs. VL+VV and allele L vs. allele V contrasts (OR = 2.72, 95% CI = 1.28–5.70; OR = 1.33, 95% CI = 1.01–1.75) (Table 2); meanwhile, a risk reducing-effect in Caucasian group under VL vs. VV (OR = 0.36, 95% CI = 0.14–0.92) (Figure 4) and LL+VL vs. VV comparisons (OR = 0.47, 95% CI = 0.24–0.93) (Table 2).

### Heterogeneity test

According to *P*-values from χ^2^-based Q-statistical test, significant heterogeneity was revealed for *SRD5A2* rs523349 (V89L) polymorphism under LL vs. VV+VL and VL vs. VV contrasts in total analysis, so the random-effects model was employed for ORs calculation in these cases while the fixed-effects model was chosen for the other three comparisons. As for the possible source of such heterogeneity, we found the significance was totally removed after subgroup analysis by ethnicity, thus indicating ethnicity might account for a part of the origin.

The fixed-effects model was used to calculate pooled ORs for *SRD5A2* rs9282858 (A49T) polymorphism due to the absence of significant heterogeneity under all genetic models.

### Sensitivity analysis

The reliability of our results was determined using sensitivity analysis. Since the number of included studies for *SRD5A2* rs9282858 polymorphism was less than 5, such analysis was not performed.

During the sensitivity examination of study on *SRD5A2* rs523349 polymorphism, no significant change in pooled ORs was detected after removal any one of individual studies, thus demonstrating that our results were robust (data not shown).

### Publication bias

**Figure 5:**
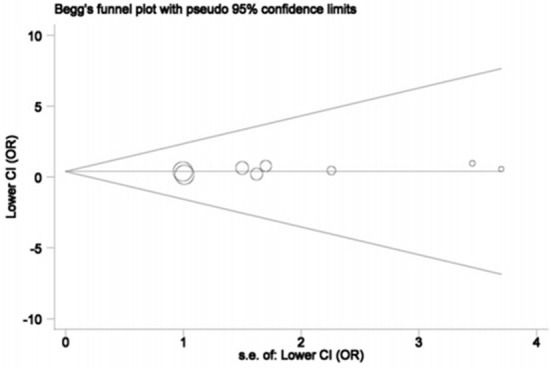
Begg's funnel plot of publication bias for *SRD5A2* rs523349 polymorphism.

The publication bias was explored using Begg's funnel plot and Egger's tests only for *SRD5A2* rs523349 (V89L) polymorphism in view of the number of included studies. The funnel plots did not show any significant asymmetry (Figure 5), and statistical evidence for the symmetry was offered by Egger's test (*P* = 0.147), manifesting the absence of significant publication bias.

## Discussion

BPH refers to the nonmalignant enlargement of the prostate induced by cellular hyperplasia, and is the major cause of LUTS in elderly males (McConnell, 1991; Stohrer et al., 2009; Nickel et al., 2010). LUTS, rather bothersome, severely interfere with the daily activities of patients (Girman et al., 1998; Welch et al., 2002). Older age is an established risk factor for BPH; studies show that this disease affects approximately 8% of males aged 31–40 years and about 80% of those aged over 80 years (McVary, 2006; Parsons et al., 2008). In the United States, an estimated $1.1 million are spent on BPH management annually (Wei et al., 2008). Therefore, it remains an urgent task to ascertain the exact etiopathogenesis of BPH so as to develop effective measures for the prevention and treatment of the disease. Major risk factors for BPH have been identified, including steroid metabolism, estrogens and androgens (Geller et al., 1976; Rohrmann et al., 2007). SRD5A2, a membrane-associated enzyme, is mainly expressed in the prostate gland and androgen-sensitive cells of the genital skin (Thigpen et al., 1992, 1993; Reichardt et al., 1995), and is responsible for the conversion of testosterone to more active DHT. Two polymorphisms rs523349 and rs9282858 have been identified in *SRD5A2* gene, and their correlation with susceptibility to prostate cancer were reported previously (Makridakis et al., 1997; Febbo et al., 1999; Nam et al., 2001). The two SNPs can affect the enzymatic activity, so their impacts on the occurrence of BPH have attracted increasing attention from investigators.

Choubey et al. investigated whether *SRD5A2* rs523349 and rs9282858 polymorphisms could affect BPH occurrence in an Indian population, and found that the VV genotype of rs523349 polymorphism was marginally related to an increased BPH risk; the rs9282858 polymorphism, however, was monomorphic (Choubey et al., 2015). In a study by Das et al., an increased BPH risk was observed in cases carrying the VL/LL genotype of *SRD5A2* rs523349 polymorphism though the association was not significant (Das et al., 2008). Nevertheless, in a study performed among Turkish men, an apparent elevation in BPH risk was found in those with the T allele of *SRD5A2* rs9282858 polymorphism, but the rs523349 polymorphism was detected to have no significant influence on the disease risk (Izmirli et al., 2011). Similarly, another study by Ersekerci et al. among Turkish men as well, showed no significant difference in genotype or allele distribution of rs523349 polymorphism between case and control groups (Ersekerci et al., 2015). A study by Li et al. found no apparent linkage between the two SNPs and BPH in a Japanese population either (Li et al., 2003).

Possible reasons explaining the above controversy may include: (1) the studied people belonged to different ethnicities; (2) selection criteria for study subjects varied among different studies, so basic characteristics such as age period and lifestyles might be statistically different; (3) not all studies obtained their results after adjustment of conflicting factors, thus possibly causing biased conclusions; and (4) some studies only had a relatively small number of participants, causing reduced statistical power of final results.

To our knowledge, this is the first meta-analysis comprehensively performed to assess the relationship between *SRD5A2* (rs523349 and rs9282858) polymorphisms and the risk of BPH. This meta-analysis, including a total of 1,865 cases and 1,424 controls, was strictly implemented from literature search to data syntheses. According to pooled ORs, *SRD5A2* rs9282858 (A49T) polymorphism increased the BPH risk under allele T vs. allele A contrast in total analysis as well as in Caucasian subgroup after stratified analysis by ethnicity. The rs523349 (V89L) polymorphism displayed no significant correlation with the disease susceptibility in total analysis; in stratified analysis based on ethnicity, it reduced the risk in Caucasian group under LL+VL vs. VV and VL vs. VV contrast while increased the disease risk in other-ethnicity group under LL vs. VV, LL vs. VV+VL and allele L vs. allele V genetic models.

The present study had certain advantages in itself, like a relatively large sample size. However, there still were some limitations to be noted when applying our findings, since meta-analysis is a secondary analysis (Zeng X. et al., 2015; Zeng X. T. et al., 2015). First of all, objects in all included studies were enrolled from hospitals, so they might not be qualified enough to represent general populations. Secondly, interfering environmental factors were not adjusted in all selected studies, thus causing certain bias in overall OR evaluations. Thirdly, gene-gene and gene-environment interactions were not analyzed due to lack of original data. Fourthly, the small number of included articles may affect the stability of the final results.

All in all, the present meta-analysis suggests that *SRD5A2* rs9282858 (A49T) polymorphism may be a risk factor for BPH occurrence, especially in Caucasians; while the rs523349 (V89L) polymorphism may present different effects on the disease onset in people of different ethnic lines.

Future studies should consider the mentioned limitations, such as selection of objects, adjustment of interfering factors and large sample size for their designing of studies. Larger-scale and better-designed studies should be conducted to verify our findings.

## Author contributions

XTZ and XHW designed this study; XJS and HW searched databases and collected full-text papers; SL and TZL extracted and analyzed data; XTZ, XJS, and SL wrote the manuscript, TZL reviewed the manuscript.

### Conflict of interest statement

The authors declare that the research was conducted in the absence of any commercial or financial relationships that could be construed as a potential conflict of interest. The reviewer KP and handling Editor declared their shared affiliation, and the handling Editor states that the process nevertheless met the standards of a fair and objective review.
